# Guidelines for designing and interpreting drought experiments in controlled conditions

**DOI:** 10.1093/jxb/erae292

**Published:** 2024-07-03

**Authors:** Menachem Moshelion, Karl-Josef Dietz, Ian C Dodd, Bertrand Muller, John E Lunn

**Affiliations:** The Robert H. Smith Institute of Plant Sciences and Genetics in Agriculture, The Robert H. Smith Faculty of Agriculture, Food and Environment, The Hebrew University of Jerusalem, Rehovot 7610001, Israel; Department of Biochemistry and Physiology, Faculty of Biology, Bielefeld University, Universitätsstraße 25, D-33615 Bielefeld, Germany; Lancaster Environment Centre, Lancaster University, Bailrigg, Lancaster LA1 4YQ, UK; INRAE-LEPSE, Institut Agro, Université Montpellier, UMR 759 Laboratoire d’Ecophysiologie des Plantes sous Stress Environnementaux, 34060 Montpellier, France; Max Planck Institute of Molecular Plant Physiology, Am Mühlenberg 1, D-14476 Potsdam-Golm, Germany

**Keywords:** Consensus-driven terminology, drought experiments, experimental design flaws, genotype×environment interactions, irrigation control, plant-environment interactions, whole-plant transpiration

As *Journal of Experimental Botany* (*JXB*) editors, we often receive manuscripts on drought tolerance and plant responses to water deficit. We have observed that the quality of research in this field frequently suffers from flawed experimental designs, inconsistent terminology, overinterpretation of data, or unrealistic lab-to-field extrapolations. To tackle these challenges, the *JXB* Editorial Board established a working group to guide better experiment design, data interpretation, and reporting of results, focusing on experiments performed in supposedly ‘controlled conditions’. Our recommendations include the following.

- Utilizing precise, consensus-driven terminology to clearly communicate objectives and hypotheses.
- Designing experiments that account for the complexities of genotype–environment (G×E) interactions, by including sufficient biological replicates, conducting multiple experiments, and measuring soil and plant water status as well as microclimate variables.
- Considering that whole-plant transpiration interacts with pot size and soil substrate to alter soil moisture and stress levels, and acknowledging that plant responses to drought depend on, and also affect, their growth dynamics.

These guidelines aim to enhance research quality, contributing vital knowledge to combat the growing threat of drought to agriculture.

## Too many drought-related papers are prone to biases and misinterpretations

Achieving agricultural sustainability is essential to meet the nutritional needs of a growing population (Scherer *et al*., 2018; Hinz *et al*., 2020). Among the threats to which agriculture is exposed, drought consistently claims first place. Despite this prominence, clear standards for conducting drought experiments are conspicuously absent. As a consequence, researchers often operate according to their own perspectives and principles, and this practice hinders reproducibility and reliable interpretation.

As the name implies, the *Journal of Experimental Botany* has always had a focus on experimental botany. Therefore, as scientific editors of the journal, we believe it is our responsibility to raise, discuss, and advocate best practices in drought experimental design. This should help both researchers and decision-makers with scientifically grounded results. As *JXB* editors, we regularly receive manuscripts that address drought tolerance issues and/or plant responses to water deficit. However, we share a concern that a significant number of manuscripts show serious flaws. Two main reasons are that: (i) the precautions necessary for unbiased experimental design and conclusions are not taken; and (ii) conclusions are overstated, particularly in relation to the significance of results obtained in controlled conditions as they relate to field performance of crops.

Here, our objective is to provide best-practice guidelines that address the research challenges specifically associated with drought experiments when they are performed in ‘controlled conditions’—in greenhouses or growth chambers which represent a large proportion of submitted papers on drought. These experiments are most often conducted in pots, where biases can occur due to improper management or characterization of drought, hampering comparisons of treatments or genotypes.

A second problem is the gap between the ambition and achievements of many drought studies. A common claim is to have identified mechanisms and processes involved in securing or enhancing crop yields under drought stress, when in reality the study was based on reductionist experimental designs with limited data collection and/or analyses of limited general relevance, potentially leading to misinterpretation of the results.

A third problem with the drought-related literature is a lack of consistency and coherence of terminology, despite well-defined terms that have been established for >50 years to characterize the different strategies plants adopt when faced with water deficit (e.g. escape, avoidance, or tolerance, see below). Moreover, the overall, and maybe overarching, issue is the lack of recognition of the fact that none of these strategies defines a drought resistance outcome, which depends on the objective and the climatic scenario. For instance, a ‘drought-avoidance’ phenotype related to early stomatal closure can be either beneficial when soil water is limited and water needs to be saved to complete the growth cycle, or detrimental if water is only lacking during limited periods (Hu *et al*., 2022). Similarly, other strategies, such as escape (e.g. early flowering) or tolerance (e.g. maintaining growth under low water potential), may have positive or negative impacts on plant survival and yield. Unfortunately, ‘drought resistance’ and ‘drought tolerance’ are often used indiscriminately. Proper use of generally accepted definitions in drought-related papers would be a major step in the right direction (Volaire, 2018). We hope that these guidelines will not only help authors to design their studies, but also help reviewers and editors to assess the robustness and transferability of results.

## Drought response assessment as a multistep process in controlled conditions

Investigations of plant responses to water deficit have followed a common path, starting from the theoretical and basic research phase, through the pre-field phase, and culminating in field trials. Field experiments test whether laboratory findings apply in real-world scenarios. Like human clinical trials in medicine, field trials represent the ultimate test for our mechanistic understanding of drought responses, but their scope and capacity are inherently limited. While acknowledging the pivotal role of field experiments in validating strategies for crop yield improvement, this Editorial addresses the basic and pre-field experimental approaches, with particular focus on drought and high-temperature stress that is often associated with drought.

Experimental botany is important during the pre-field phases, specifically in pot-based studies conducted in growth chambers and greenhouses with varying levels of environmental control. These studies facilitate systematic hypothesis testing and the dissection of complex mechanisms. By enhancing the experimental methodology during these foundational stages and accurately describing the growth conditions, we aim to foster more effective, efficient, and well-informed field studies in the future.

**Fig. 1.**
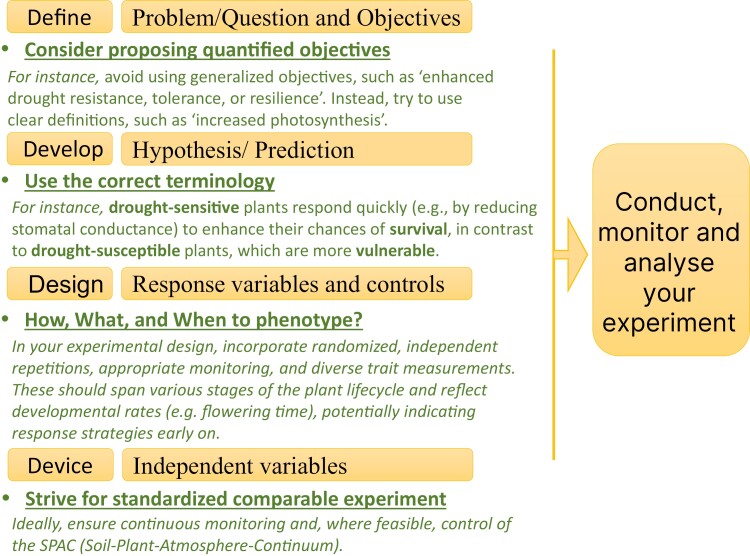
Sequential framework for designing and conducting plant drought experiments: the four ‘D’s checklist approach. To ensure scientific rigour and reproducibility of stress experiments (drought in particular), we propose the following four sequential steps. Firstly, defining the overarching research question or problem is crucial in any research endeavour. A classical question reflecting the issue discussed in this paper might be: ‘Can we improve yield production under drought stress?’ Following this, when defining the research objective, we recommend avoiding overly general terms (e.g. ‘improving plant drought tolerance’), and instead aim for more specific and quantitative objectives (e.g. ‘minimizing the yield penalty caused by drought’, or ‘understanding the molecular mechanisms regulating root length for reaching deep water’). This precise and, ideally quantitative, definition streamlines the subsequent stages, particularly hypothesis development. Furthermore, it also aids in conclusively determining, during the discussion phase, whether the study has achieved its research goals. The second stage involves developing specific, testable hypotheses or predictions. For instance, the hypothesis could be: ‘the cultivars with long roots will reach a deeper water table enabling the crop to maintain water uptake, thereby yielding better under the drought period compared with cultivars with short roots’. Another example would be. ‘Gene X expressed in the guard cells will increase stomatal sensitivity to ABA, thereby inducing faster closure at less negative soil water potential, thus enhancing plant survival’. In the latter example, special attention is needed for pleiotropic effects on leaf area or flowering time, both of which directly impact the targeted process (transpiration) yet via totally different mechanisms. In the third stage, the experiment should be designed to measure all key environmental variables as well as the biological variables of interest throughout the experiment period. The final stage selects appropriate monitoring devices/probes and their arrangement. Importantly, considering the dynamic nature of the G×E interactions, the variables of interest should be monitored as often as needed to allow unbiased interpretation of the results over the whole duration of the experiment.

This Editorial advocates classical methodical investigation, with particular emphasis on plant stress biology studies, in a strategy outlined here in four ‘D’s: ‘Define, Design, Develop, and Device’ (Fig. 1, and explained thereafter). Such a structured approach might assist researchers in accounting for the complex experimental dynamics.

## Defining the research objective(s)

Precisely defining a research objective should be the first step in any study. For drought stress experiments, the performance or fitness of a crop or wild-type plant in its agronomic or natural environment should be evaluated with measurable criteria. For several decades, definitions describing a plant’s response to stress have been relatively broad. Levitt, a pioneer in the standardization of plant stress responses, suggested quantitative definitions that characterize plant reactions to stress in a more general way (Levitt, 1980). Nevertheless, many studies use the term ‘stress’ very broadly, which necessitates a more precise and quantitative definition (see Gaspar *et al*., 2002; Kranner *et al*., 2010), especially if the research objective is to minimize yield penalties under conditions of drought stress or to enhance survival under more severe conditions.

In studies of stress responses in annual crop plants, vague or oversimplified research objectives such as: improving the plant’s ‘tolerance’, ‘resistance’, or ‘resilience’ to stress are all too common and generic (Box 1). Poorly defined research objectives impede the researcher by producing unfocused research, and confuse the reader with ambiguous information. For instance, desert plants known for their drought survival and high water-use efficiency (WUE; the ratio of carbon fixation, or biomass gain, to water loss, or transpiration) are often hypothesized to have advantageous traits to manage various environmental stressors (Nobel, 1988). However, these traits, while enhancing survival, are often associated with slow growth that causes low productivity and might not align with agronomic objectives aimed at optimizing yield in annual crops under milder stress. Thus, clearly defining research objectives is essential, as it helps distinguish between strategies focused on survival in harsh conditions and those aimed at maintaining yield under milder stresses. Such differentiation is key to tailoring research and interventions that meet specific agronomic or ecological goals.

By establishing clear objectives, researchers gain insights into the intricate interactions between the plant and its environment, identifying genetic, epigenetic, and environmental factors contributing to observed responses. Defining the objective helps us to understand the complex interactions and adaptations that occur within ecosystems or field conditions. This analysis, however, is only as reliable as the shared language and concepts we use to frame our research questions. As we push the boundaries of our knowledge, it is essential that we ensure consistent and uniform terminology, similar to standards in physics.

## Developing research hypotheses using consensus terminology

Deciphering plant responses to environmental stressors is not straightforward. The response network depends on evolutionary adaptations, survival strategies, and productivity trade-offs. To advance our understanding of these mechanisms, developing testable research hypotheses and predictions, in accordance with the research goal (see above), is critical. Hypotheses or predictions are not only essential for developing the proper experimental design, but they should also be explicitly outlined when publishing the study.

Use of precise definitions and consensus terminology in stress biology research is essential throughout all stages of the research process. Observing this principle from the very beginning of a study helps the researcher to define hypotheses that are not only concise and logically coherent, but also capable of being tested and understood. For example, terms such as ‘sensitive’ and ‘resistant’ are often used to denote opposite behavioural definitions. However, in practice, plants that are more sensitive to environmental signals tend to respond more quickly, thereby enhancing their chances of ecological resistance. Consequently, in the context mentioned above, the term ‘susceptible’ would more accurately describe plants that are damaged by stress. The following examples illustrate the use of consensus-driven terminology: plants that are sensitive to early-stage stress signals will probably reveal more defensive response (e.g. stomatal closure with decreased soil water availability), thereby improving the plant’s chances of survival (e.g. staying alive regardless of its size or reproductive capabilities). Conversely, a delayed response (e.g. slower and later stomatal closure with decreased soil water availability) may prolong the biochemical and physiological activity, for example photosynthesis, but exposes plants to higher risks of detrimental dehydration, reducing their survival chances. Therefore, stress sensitivity is a desirable ecological resistance mechanism when the objective is ‘not to die’, while stress insensitivity may enhance short-term agronomic (or yield) tolerance (e.g. maintaining production despite the development of the stress). While stress insensitivity may enhance yield under mild to moderate conditions, it will result in plant susceptibility to the stressor with prolonged stress periods. Thus, this trait (stress insensitivity) might be beneficial under mild to moderate stress, but makes the plant susceptible (the likelihood of being adversely or even lethally affected) to more severe stress (see more in Tardieu *et al*., 2018). Sensitivity to stress can activate stress avoidance mechanisms, indirectly helping plants evade stress rather than directly coping with it. These mechanisms include stomatal closure, developing longer and deeper roots to access groundwater, early flowering to escape the stress period, or desiccation tolerance as seen in resurrection plants. However, these mechanisms often lead to changes in resource allocation, potentially affecting future growth and reproduction, and consequently long-term productivity and yield (in agriculture). This raises questions about their agronomic advantages.

Box 1. **Defining resilience: precision in plant stress response terminology**

To accurately assess the efficacy and overall capacity of several **stress defence mechanisms**, it is valuable to measure the plant’s recovery rate or its **stress resilience**—the plant’s ability to recover from stress (Moshelion, 2020; Ingrisch *et al*., 2023). This recovery rate (for example, in daily transpiration or biomass gain) reflects the cumulative activity and efficacy of the plant’s stress defence mechanisms throughout the stress exposure. Measuring the duration and intensity of maximal soil water deficit before recovery is essential to interpret the ability of different genotypes to recover. A rapid recovery suggests efficient stress defence mechanisms that maintain essential cellular processes, limit damage (e.g. preventing embolism or root desiccation), and enable prompt repair after stress. Conversely, slow recovery implies less efficient defences, indicating more extensive stress-induced damage or less efficient activation of repair and a longer recovery period.

## Designing effective experiments in G×E research

Understanding the intricate interplay between plants and their environment presents a demanding experimental challenge, due to the multiplicity and variability of environmental factors [e.g. light, temperature, vapour pressure deficit (VPD), and soil water content] and the diverse biological responses they elicit (Zandalinas and Mittler, 2022). This complexity poses a challenge to the researcher when designing experiments and interpreting the data.

**Fig. 2.**
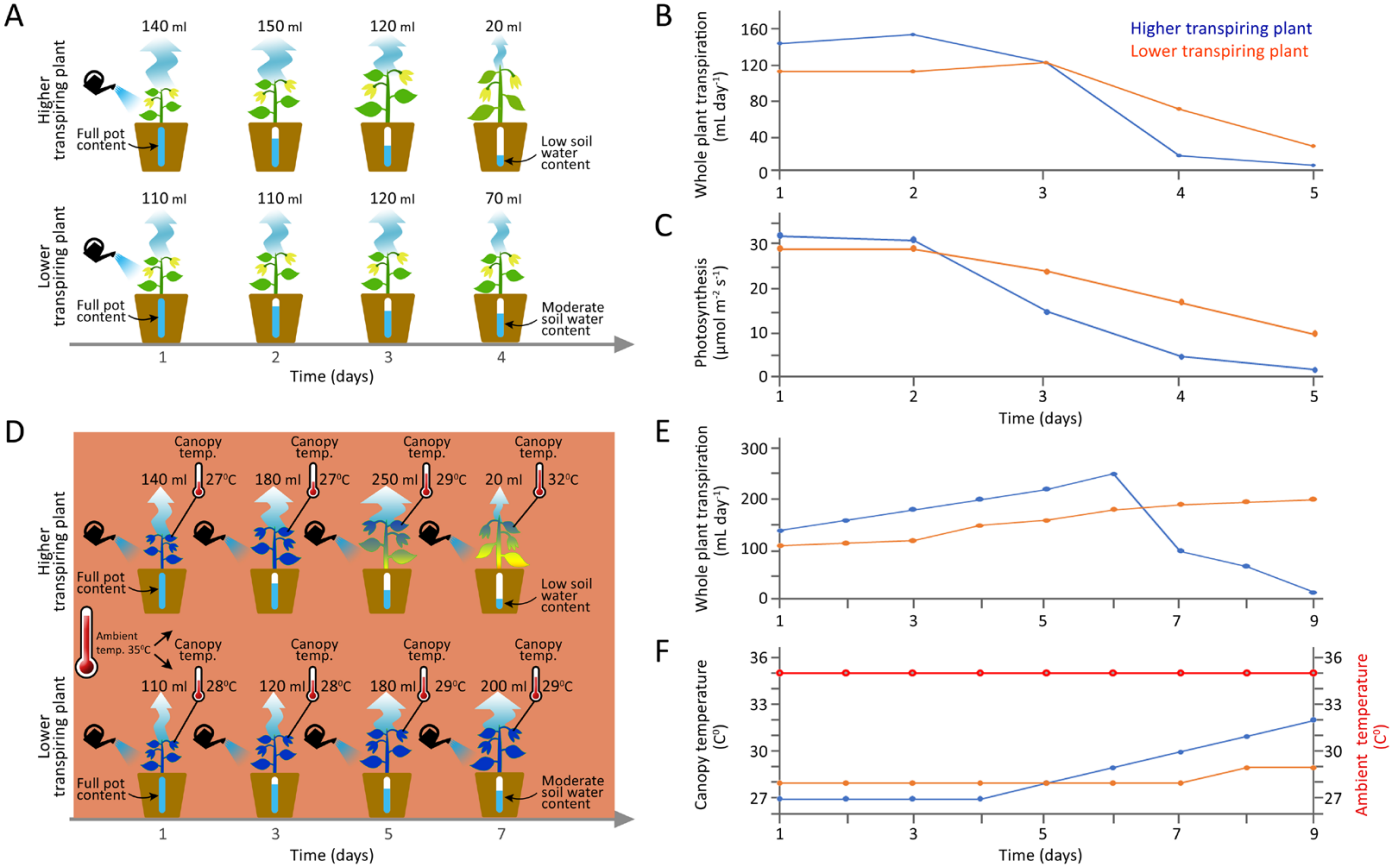
Unintended bias in the experimental design and stress analysis studies. The schematic diagram illustrates common experimental pitfalls. In pot experiments, the most common unintended biases result from a lack of awareness regarding the pot water content (i.e. the difference between plant transpiration and the water available to it in the pot). Usually, these experiments aim to apply equivalent stress treatment levels across several genotypes. However, differences in transpiration result in unequal exposure of plants to varying soil water content. Here, we highlight two common challenges. (A) Unintentional differential drought experiment: this experiment was designed to assess the drought responses of two genotypes. After establishing plants in well-watered conditions, the soil in the pots was allowed to dry at rates determined by the plants’ own transpiration and soil surface evaporation, without any feedback irrigation control. However, this experimental design does not subject the genotypes to similar stress levels; instead, it heavily emphasizes spatial and biological variations between plants. As a result, cultivars with higher transpiration rates (A, top panel), which are also likely to assimilate more CO_2_ and thus grow faster, will experience much faster and more severe stress compared with those with lower transpiration rates (A, lower panel). Notably, this variation differentiates the cultivars. However, it is a function of transpiration rate, not a drought response. Moreover, it fails to adequately compare the two lines under the same stress level since the plants with lower transpiration rates were not subjected to drought conditions equivalent to those with higher rates. Therefore, highly transpiring plants are expected to unintentionally experience a faster rate of soil water depletion. This differential stress can subsequently alter some important physiological traits. These include (B) daily whole-plant transpiration and (C) momentary photosynthetic rate (both graphically visualizing ‘A’, where day 1 is marked as the final irrigation day). Another example of an unintended stress artefact experiment involves heat stress, which is characterized by exposure to high air temperatures. (D) Thermal image demonstration of unintentional differences in canopy temperature under a heat experiment. Throughout this experiment, all plants were exposed to a heated atmosphere (35 °C) and watered daily. However, plants with higher transpiration rates (D, top panel) maintained a lower canopy temperature than those with a lower transpiration rate (D, lower panel) during the initial days of the experiment. Thus, better growth and increased transpiration of these plants could inadvertently lead to faster (yet unexpected) soil water deficit. In turn, this drought side effect causes further genotypic variation in canopy temperatures that deviate from the desired experimental conditions [as graphically visualized in (E) daily whole-plant transpiration and (F) canopy temperature versus ambient air temperature]. Consequently, one could mistakenly conclude that the plants with lower transpiration are ‘heat-resistant’, even though they were not exposed to stress conditions (both drought and heat) similar to the high-transpiring plants. It is important to note that transpiration is highly correlated with CO_2_ assimilation. Thus, these experimental protocols (A and D) may favour plants with lower transpiration and thus lower production (ecological resistance, as explained above). An additional major pitfall, potentially resulting from the biased experimental design described above, pertains to the conduct of omics studies: there is a risk of confounding causes and consequences.

The issue can be more complex as some environmental conditions can depend on other traits, such as soil water status that depends on transpiration. Moreover, comparing genotypes with different whole-plant transpiration levels under the same environmental conditions will lead to genotype differences in soil moisture levels. While researchers aim to subject various genotypes to equivalent stress, genotypic differences in whole-plant transpiration rates cause differential rates of soil drying. Consequently, without feedback irrigation control, it becomes challenging to subject different genotypes to similar degrees of stress (see Fig. 2 for an illustration).

Nevertheless, even when working with the same genotype, spatial and temporal variations can still lead to inconsistencies in stress application, due to differences in soil drying rates. While plants are often grown in greenhouses to provide some environmental regulation, closer examination reveals a multilayered, multifactorial complexity. For instance, daily variation in sunlight causes fluctuating temperature and relative humidity, which in turn dynamically affects the VPD that plants experience (Shamshiri *et al*., 2018). This temporal environmental shift, influenced not only by the daily solar cycle but also by seasonal and weather conditions, presents a perpetually changing scenario for the plants. Furthermore, the position of a plant within the greenhouse can significantly modify its individual microenvironment, due to variations in sunlight exposure, airflow, and other factors. Consequently, plants encounter substantial temporal and spatial variations within the greenhouse, necessitating a holistic approach and/or models that account for these variations (Cabrera-Bosquet *et al*., 2016).

Capturing these interactions poses a challenge due to plant responses to their dynamically changing environment. However, the experimentalist is often limited in terms of plant material, sample number, analytical capacity, and available resources. Therefore, we strongly recommend that researchers: (i) recognize and report the limitations in their experimental design; (ii) take steps to assess the impact of complex environmental interactions (e.g. comprehensive soil-–plant–atmosphere monitoring); and (iii) take account of G×E interactions when interpreting their data.

### Accounting for feedback and spatio-temporal factors in experimental design

Considering plant feedback mechanisms in response to environmental variables is crucial for meaningful, reproducible experiments. Soil-borne stresses, such as drought or salinity, occur naturally and gradually. Furthermore, feedback modulation of experimental conditions, where the output or result of a process influences the rate or extent of the same process, must be considered. For instance, microbes can change the chemical conditions of their environment or plants can alter their soil composition (Lozano *et al*., 2022). Thus, the experimental design should allow regular monitoring and (if possible) control soil water status and other related climatic conditions throughout the entire experiment, which may prevent unintentional errors and pitfalls.

Since plant–environment interactions are dynamic, careful consideration must be given to designing the experiment and the analysis. Hence, the importance of time scale in planning these dynamic experiments is paramount, primarily due to different feedback effects in response to various environmental conditions. To ensure accurate analysis when studying plant responses, it is crucial to standardize harvest times to minimize diel and circadian effects and account for variations in phenology, such as flowering time, by implementing both developmental and chronological controls during harvesting (Hotta, 2021).

Another highly important element when designing a stress experiment concerns the pot effect, influenced by the duration of the stress. Measuring all plants only at a single point in time (common to many experiments) pre-supposes that plants within a treatment group experience the same level of stress simultaneously compared with the control treatment. However, biological differences in stomatal conductance and/or vegetative development, and spatial variations in ambient conditions (position effect) cause variability in transpiration and thus different stress exposure (different levels of soil water deficit). As the pot volume is constant, any differential water losses expose the plants to different levels of stress. This distortion of results may then reflect a ‘pot effect’ rather than biological differences (Fig. 2A). As a result, comparing samples (and therefore genotypes) highlights the consequences of different transpiration rates, rather than causing them. This is a very common pitfall and a major cause of rapid rejection of drought stress manuscripts by *JXB*.

Another common, unintentional pitfall in experimental design is heat stress bias. This occurs when individuals within a treatment group experience different temperatures from one another, despite all being exposed to the same ambient temperature. Since plants can significantly cool themselves through transpiration (Maes and Steppe, 2012), reporting only ambient temperature but not canopy temperature can result in incomplete data that inadequately explain plant responses to heat stress. Consequently, despite all plants being exposed to the same chamber temperature, their actual biological responses could vary substantially due to differences in canopy temperature. Hence leaf, canopy, or other relevant organ temperatures should be measured and reported (Fig. 2B). Moreover, heating the plant’s ambient atmosphere reduces the relative humidity, thereby increasing VPD. With current technologies it is still a major challenge to modulate ambient temperature and relative humidity in growth chambers without altering the VPD, which can introduce an additional (and often unaccounted for) stress factor. At the very least, VPD should be monitored and reported, and its relevant side effect impact on transpiration should be discussed.

Strategies such as using larger, heat-insulated pots to prevent root restriction (Poorter *et al*., 2012) and root warming, and a homogenous soil (within and between pots within the same treatment group) help to mitigate pot-related effects. Larger pots and more porous substrates facilitate better drainage to encourage root proliferation, although anoxic layers may still occur at the base (Passioura, 2006), requiring careful irrigation to prevent overwatering. Surface irrigation to a target pot weight may result in substantial vertical soil moisture gradients within the pot, which can attenuate plant responses to soil water availability (Puértolas *et al*., 2017). While some of these issues are best managed by carefully selecting pot size according to the expected experimental duration, reporting soil physical properties such as soil moisture release curves and soil/plant water status measurements will help interpret the experimental results. Lastly, but importantly, thorough and precise reporting of all measured variables is necessary, as only the availability of these data ensures the validity and reproducibility of the experiment.

From a practical standpoint, achieving standardization in stress experiments requires careful consideration of several fundamental questions. How is the experimental setup structured and how is the independent variable maintained? Which biological parameters are being measured? At what time points should these parameters be measured to accurately compare different genotypes and control groups? Addressing these questions is essential to alert authors to potential pitfalls and ensure accurate measurement of plant stress levels. Certainly, other important biological considerations will arise, such as the developmental stage during sampling and the genetic relationship of the compared germplasm, among other factors specific to the research question. While these biological aspects warrant careful consideration, they do not alter the fundamental principles addressed in the ensuing discussion. Therefore, while acknowledging their importance, we will elaborate on these specific biological issues and questions in the following.

## Device utilization to maintain and monitor desired stress levels in experiments

Maintaining the desired experimental conditions presents a significant challenge, particularly if striving for consistent application of stress levels (defining and controlling independent variables) to all plants throughout the entire experimental period, despite differences in genetics and location within the greenhouse.

To effectively address this issue, one can utilize either simple or more advanced technologies. These can be used to differentially regulate each plant’s conditions to meet the stress level requirements. For instance, a simple solution for comparing two cultivars under similar water stress conditions (as depicted in Fig. 2A) could involve planting them in the same pot. This ensures that they are exposed to identical local stress levels concurrently and continuously. However, such a mixed culture may cause below-ground competition and interactions, and elicit other feedback processes that are outside the focus of the experiment.

More technologically advanced solutions are also available. These include continuously measuring each plant’s transpiration and soil water content, coupled with automatic feedback regulation of each pot’s water content in relation to the plant demand (Negin and Moshelion, 2017; Gosa *et al*., 2019). Such technologies are already available in automated phenotyping facilities and can accommodate a large number of plants, thereby facilitating high-throughput experiments (Granier *et al*., 2007; Sadok *et al*., 2007; Tester and Langridge, 2010; Dalal *et al*., 2020). For a limited number of pots, such control measurements and soil water adjustments can also be achieved manually.

### What to measure?

In any plant stress experiment, the measured variables (dependent variables) should be selected according to the specific requirements of the experiment. These variables can cover molecular, biochemical, physiological, and morphological aspects. Measuring these variables should avoid subjective estimates by the researcher (e.g. plants looking ‘stressed’ or wilted). Visual images may be useful to illustrate the impact of the stress on the overall appearance of the plant, but on their own can lead to superficial and incorrect conclusions. Therefore, quantitative data, such as those related to plant and soil water status, are needed as a robust foundation for statistical analysis, along with a rigorous interpretation of the phenotype.

However, it is essential to understand that plant responses to stress are hierarchical in nature (Hsiao, 1973). Thus, some parameters, such as leaf elongation rate (Sadok *et al*., 2007) and stomatal conductance, or specific molecular and biochemical markers (e.g. abscisic acid or redox imbalances), are highly sensitive and respond swiftly to minor changes in stressors (Hilty *et al*., 2021; Dietz and Vogelsang, 2023). Conversely, parameters such as plant biomass or leaf colour may take longer to respond.

It is also important to recognize that morphological changes can be misleading as stress markers. For example, a less turgid leaf might appear stressed, but it could actually indicate an anisohydric productive behaviour of maintaining transpiration and photosynthesis instead of closing stomata to preserve turgor loss which could signify a ‘risk-taking’, productive plant (Sade *et al*., 2009). As such, morphological changes should not be considered the primary indicators of stress impact. Moreover, the morphological manifestations of plant stress responses can be multifaceted, varying based on factors such as plant species, developmental stage, and environmental conditions.

In contrast, incorporating physiological measurements, such as growth, transpiration, and water potential, is strongly recommended to determine stress levels (Muller *et al*., 2011). These parameters are higher in the response hierarchy and are usually the first to change.

### When to measure?

It is important to remember that the above physiological parameters are spatially and temporally variable. When deciding which traits to measure and how often, the researcher should consider their intrinsic spatio-temporal variations, along with (ideally) continuous observations of ambient conditions. Technological developments allow more extensive monitoring of plant and environmental parameters than in the past, and the commercialization and falling costs of sensors bring this goal within reach for many researchers (Gosa *et al*., 2019).

Determining the appropriate frequency and specific time points for measurements, particularly when stress begins, poses another challenge in plant stress experiments. To comprehensively understand plant stress responses, it may be advisable to conduct a more detailed study that includes pre-stress, stress, and post-stress response profiles, alongside a control group not subjected to soil water deficit or increased canopy temperature. This design ensures that any observed changes can be specifically attributed to the stressor, and not to natural variations or other factors. It also facilitates an understanding of how the plant reacts to, copes with, and potentially recovers from stress over time.

As previously mentioned, due to the dynamic nature and feedback modulation of experimental conditions, sampling based solely on the time since applying a stressor is very likely to lead to inaccurate comparisons, for example soil water content, whole-plant transpiration, photosynthesis, etc. (as illustrated in Fig. 2). Determining when stress begins should be based on plant response. For example, determining the point when transpiration starts to decline should indicate the beginning of drought stress, as it signifies that soil water availability has become insufficient to support the previous rate of transpiration.

The potential solution to this challenge is to carefully select traits and their measurement frequency, considering spatio-temporal characteristics, as mentioned earlier. Ultimately, there is no one-size-fits-all approach for studying plant stress responses. Selecting variables to measure and interpreting results must be tailored to the experimental goals and context. Nevertheless, adhering to the principles suggested above will significantly enhance the experimental design, conduct, analysis, and repeatability of the study.

The aim of this editorial opinion article is to alert experimentalists exploring drought (and related stress such as elevated temperature) effects on plants to some of the pitfalls in this field, and thereby help them to design the optimal experiment for the given purpose. Our main recommendations are as follows: (i) to define the overall objective in precise terms; (ii) to develop a meaningful and testable hypothesis; (iii) to design experiments that will allow robust and unambiguous testing of the hypothesis; and (iv) to utilize protocols to optimize the monitoring and control of environmental conditions, thereby enhancing the precision and reproducibility of stress experiments. This approach not only boosts the reproducibility of experiments but also improves the interpretability of the data. We recognize that logistical and financial constraints can require compromises in the experimental design, such that not all recommendations can be met. This does not necessarily preclude publication of the work; however, in such cases it is essential that the data interpretation and discussion reflect the limitations in the experimental design. Drought is an ever-present threat to natural ecosystems and agricultural production, and its adverse impact on both is likely to increase with global climate change. The plant science community is at the forefront in meeting this challenge, but success in mitigating the impact of drought on plant life will depend on a proper understating of plant responses to drought. We hope that our recommendations for the rigorous design, execution, interpretation, and reporting of experiments will help plant researchers rise to this challenge.
